# Retraction: miR-135a inhibits malignant proliferation and diffusion of non-small cell lung cancer cells by down-regulating ROCK1 protein

**DOI:** 10.1042/BSR-2020-1276_RET

**Published:** 2021-09-22

**Keywords:** miR-135a, non-small cell lung carcinoma, ROCK1

This article is being retracted from *Bioscience Reports* at the request of the authors following receipt of a notification from a reader, alerting the Editorial Office to unusual data presented in Figures 2 and 3.

The Editorial Office requested the raw data from the authors and this has not been provided. The authors wish to retract the article. The Editor-in-Chief and Editorial Board agree with the Retraction.

